# The dynamic switch mechanism that leads to activation of LRRK2 is embedded in the DFGψ motif in the kinase domain

**DOI:** 10.1073/pnas.1900289116

**Published:** 2019-07-10

**Authors:** Sven H. Schmidt, Matthias J. Knape, Daniela Boassa, Natascha Mumdey, Alexandr P. Kornev, Mark H. Ellisman, Susan S. Taylor, Friedrich W. Herberg

**Affiliations:** ^a^Department of Biochemistry, University of Kassel, 34132 Kassel, Germany;; ^b^National Center for Microscopy and Imaging Research, University of California San Diego, La Jolla, CA 92093;; ^c^Department of Neurosciences, University of California San Diego, La Jolla, CA 92093;; ^d^Department of Pharmacology, University of California San Diego, La Jolla, CA 92093

**Keywords:** kinase architecture, LRRK2, Parkinson’s disease, DFG motif, Leucine-rich repeat kinase 2

## Abstract

Leucine-rich repeat kinase 2 (LRRK2) is a large multidomain protein, and LRRK2 mutants are recognized risk factors for Parkinson’s disease (PD). Although the precise mechanisms that control LRRK2 regulation and function are unclear, the importance of the kinase domain is strongly implicated, since 2 of the 5 most common familial LRRK2 mutations (G2019S and I2020T) are localized to the conserved DFGψ motif in the kinase core, and kinase inhibitors are under development. Combining the concept of regulatory (R) and catalytic (C) spines with kinetic and cell-based assays, we discovered a major regulatory mechanism embedded within the kinase domain and show that the DFG motif serves as a conformational switch that drives LRRK2 activation. LRRK2 is quite unusual in that the highly conserved Phe in the DFGψ motif, which is 1 of the 4 R-spine residues, is replaced with tyrosine (DY^2018^GI). A Y2018F mutation creates a hyperactive phenotype similar to the familial mutation G2019S. The hydroxyl moiety of Y2018 thus serves as a “brake” that stabilizes an inactive conformation; simply removing it destroys a key hydrogen-bonding node. Y2018F, like the pathogenic mutant I2020T, spontaneously forms LRRK2-decorated microtubules in cells, while the wild type and G2019S require kinase inhibitors to form filaments. We also explored 3 different mechanisms that create kinase-dead pseudokinases, including D2017A, which further emphasizes the highly synergistic role of key hydrophobic and hydrophilic/charged residues in the assembly of active LRRK2. We thus hypothesize that LRRK2 harbors a classical protein kinase switch mechanism that drives the dynamic activation of full-length LRRK2.

## Significance

Little is known about the regulation of Leucine-rich repeat kinase 2 (LRRK2) associated with familial Parkinson’s disease (PD). To test whether the kinase domain drives LRRK2 activation, we applied the spine concept that describes the core architecture of every protein kinase. We discovered that mutation of Y2018, a regulatory spine residue, to Phe in the DFGψ motif created a hyperactive kinase similar to the PD-associated mutation G2019S. The hydroxyl moiety of Y2018 thus serves as a “brake,” stabilizing the inactive conformation; simply removing it destroys a key inhibitory hydrogen-bonding node. These data reveal an LRRK2-specific regulatory mechanism, confirming that the kinase domain functions as a classical kinase that controls overall conformational dynamics in full-length LRRK2 and drives therapeutic strategies.

**Fig. 1.**
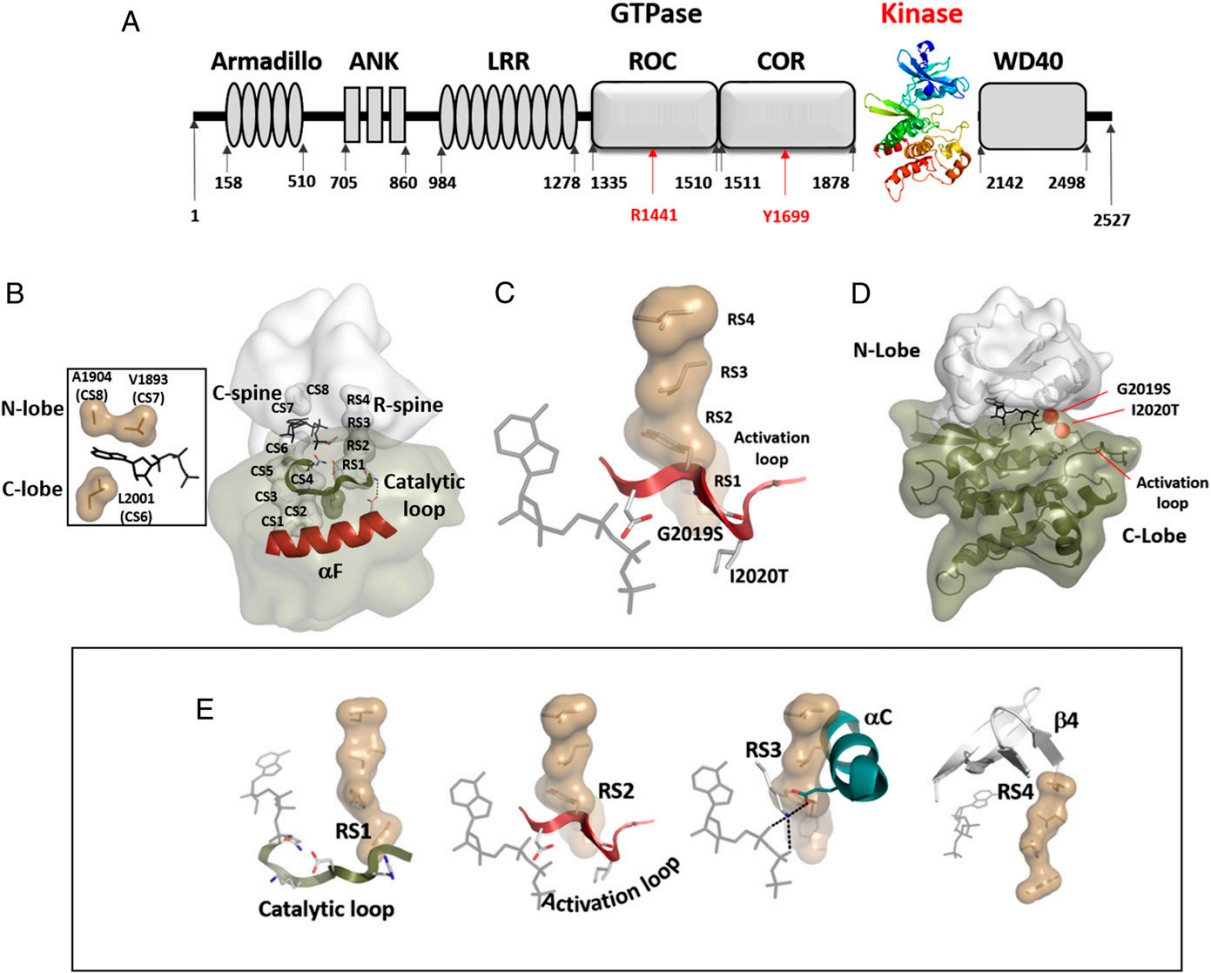
Identification of kinase spines in LRRK2. (*A*) Domain composition of LRRK2 highlighting the kinase domain as a central hub for LRRK2 regulation. (*B*) Spine residues in LRRK2 were identified based on a structural model of SRC1. The C-spine (CS1 to CS8) and the R-spine (RS1 to RS4) are connecting the N lobe (light gray) and the C lobe (dark green) and are bridged via the αF helix (red). The C-spine can only be completed by the recruitment of ATP (*Left*). (*C* and *D*) The magnesium-positioning loop contains the DYGψ motif, which encompasses 2 of the most common PD-related mutations (G2019S and I2020T). This loop is a hotspot for disease mutations, with D2017 being essential for orientating the γ-phosphoryl of ATP in most protein kinases. (*E*) The R-spine connects several highly conserved motifs in the kinase core. RS1 is part of the catalytic loop, including the HRD motif (YRD in LRRK2), while RS2 is an anchor of the AL. RS3 is orientating the αC helix. The highly flexible DYG motif as well as the αC helix are stabilized by R-spine formation via hydrophobic interactions between RS2, RS3, and RS1, with RS4 belonging to the β-strand 4 of the N lobe.

Parkinson’s disease (PD) is one of the most prevalent neurodegenerative diseases, and proper treatment is not available due in large part to our poor understanding and contradictory data regarding the underlying pathogenic mechanism (1–3). One gene product identified in PD pathogenicity is the Leucine-rich repeat kinase 2 (LRRK2) encoded on the *PARK8* gene. LRRK2 is a large multidomain protein (Fig. 1*A*) that contains several mutations that are known to be important risk factors for familial PD (4–7). Although cross-talk between GTPase domains and kinase domains are frequent in biology, LRRK2 is one of the few examples in the human kinome where both domains are embedded in the same polypeptide chain (8, 9). While the precise mechanism for LRRK2 activation and function is still being explored, it is thought that LRRK2 exists in several different conformational states. In the cytoplasm, it is predicted to be an inactive monomer, while an active dimer/multimer is present at membranes (10, 11). In addition, 3 of the 5 most common pathological, familial mutants of LRRK2 (R1441C, Y1699C, I2020T) are predicted to form filamentous structures that represent LRRK2 docked onto microtubules (12). Until now, it is not clear if the docking onto microtubules is associated with the pathogenicity of those mutations. However, these results indicate that conformational flexibility is thus an intrinsic feature of LRRK2. LRRK2 is also a protein kinase A (PKA) substrate, and phosphorylation promotes docking of LRRK2 onto 14-3-3 proteins (13–17). Finally, Rab proteins are known substrates of LRRK2 (18–22). LRRK2 thus has many docking partners and interfaces. Our goal here is to explore how the conformational dynamics of the kinase domain contribute to the overall conformational dynamics of full-length LRRK2.

The kinase core of LRRK2 is depicted in Fig. 1 and *SI Appendix*, Fig. S1. In summary, there are 2 hydrophobic “spines” that define the core architecture of every eukaryotic protein kinase (23–25). The first to be recognized was the regulatory (R) spine that is assembled in every active kinase but typically broken in inactive kinases (23, 25). Protein kinases, like G proteins, are dynamic molecular switches, and the essential elements for activating the switch mechanism are embedded in the assembly of the R-spine (23). In addition, there is a catalytic (C) spine that is completed by the adenine ring of adenosine triphosphate (ATP), and occupancy of this pocket synergizes the kinase for transfer of the phosphate (24). The R-spine (RS) and C-spine (CS) residues of LRRK2 as well as the positions of the 2 familial kinase domain mutations, G2019S and I2020T, are summarized in Fig. 1 *B* and *D*, and the nomenclature for the spine residues is in Table 1 and *SI Appendix*, Fig. S1.

**Table 1.** Numbering and comparison of PKA, LRRK2, and BRAF spine residues

Motif and numbering	PKA	LRRK2	BRAF
C-spine			
CS8	A70	A1904	A481
CS7	V57	V1893	V471
	ATP	ATP	ATP
CS6	L173	L2001	F583
CS5	I174	L2002	L584
CS4	L172	V2000	I582
CS3	M128	L1955	L537
CS2	L227	L2062	V645
CS1	M231	I2066	L649
R-spine			
RS4	L106	L1935	F516
RS3	L95	L1924	L505
RS2	F185	Y2018	F595
RS1	Y164	Y1992	H574
“Shell”			
Sh3	M118	L1945	I526
Sh2	M120	M1947	M528
Sh1	V104	I1933	L514
Regulatory triad			
RT3	D185	D2017	D594
RT2	E91	E1920	E501
RT1	K72	K1906	K483
Hydrophobic network			
HN1	L74	F1908	L485

Sh, shell residues.

To describe the core architecture of the kinase domain of LRRK2, we used BRAF and PKA as model systems. Our previous studies with BRAF (26–28) as well as subsequent NMR studies with PKA (29) validated the hypotheses that the C-spine and R-spine are critically important for catalysis and activation, respectively, and established these spines as key conserved features of protein kinase core architecture. The numbering of the corresponding residues in BRAF, PKA, and LRRK2 is included in Table 1. A481 in BRAF (A1904 in LRRK2; CS8) is a highly conserved C-spine residue that lies on top of the adenine ring. When mutated to Phe in BRAF (A1904F), ATP binding is abolished, presumably because the phenyl ring occupies the adenine binding pocket (26, 28). Although this mutant has the phenotype of a dead pseudokinase, it can, nevertheless, still bind to wild-type (wt) BRAF and/or CRAF and serve as an activator (28). Mutation of the R-spine residue L505F (RS3; L1924F in LRRK2) and the flanking residue L485F (F1908 in LRRK2) to increase hydrophobicity led to constitutive activation of BRAF (26, 27). Since LRRK2 lies on the same branch of the kinome tree and is a close homolog of BRAF, we used these 2 strategies, fusing the C-spine and altering the hydrophobicity of the R-spine, to test the importance of the R- and C-spine residues of LRRK2 for activation and conformational transitions. We used 3 different kinase assays (autophosphorylation, phosphorylation of a peptide substrate [LRRKtide], and phosphorylation of 2 different proteins [Moesin and Rab8a]). In addition, cell-based assays were used to test the effect of all mutations on protein redistribution and filament formation (12).

Using A1904F (CS8) as a prototype for a C-spine fusing mutant and multiple mutations of R-spine and R-spine flanking residues, we explored the hydrophobic space of the kinase core domain of LRRK2. In addition, we investigated the motifs that anchor the R-spine residues within the kinase core (Fig. 1*E*). Three conserved kinase motifs (the His Arg Asp [HRD] motif, the DFGψ motif [where ψ is a hydrophobic amino acid], and the αC helix) are critically important for ATP and substrate orientation as well as to prime the kinase for phosphoryl transfer. The HRD motif includes RS1 and is anchored to the catalytic loop, the DFGψ motif includes RS2 and is anchored to the Mg positioning loop, and the αC helix includes the E1920 motif and RS3, while RS4 is embedded in the β4 strand. The C-spine residue A1904 is anchored in β3 (CS8). The RS2 site was of particular interest, because 2 of the common familial mutations are located in this motif. By mutating each of the DFGψ motif residues, we discovered that the Y2018 is critical for stabilizing the inactive conformation of LRRK2. Although this residue is conserved as a Phe in most other kinases, in LRRK2, it is a Tyr. Mutating Y2018 to Phe created a hyperactive form of LRRK2, similar to but even more active than the familial G2019S mutant. The hydroxyl moiety of Y2018 thus serves as a “brake” that stabilizes an inactive conformation; simply removing it destroys a key hydrogen-bonding node. The DFGψ motif is the most highly mutated region in the cancer kinome (30), and we delineate here the unique features of each residue in this motif. Our results suggest furthermore that destabilizing the inactive state of this motif is what drives the pathogenic mutations in this region. Both Y2018 and I2020 are critical for locking LRRK2 into an inactive state. Here, we define the presumed docking sites for these 2 residues, one most likely driven by hydrogen bonding and the other driven by hydrophobicity, as essential for stabilizing the inactive conformation of LRRK2.

## Results

### Identification of Spine Residues in the Kinase Core of LRRK2.

Hydrophobic spines are critical hubs in the core of every kinase. In addition to their role in orienting residues for the catalytic step (i.e., phosphoryl transfer), formation of these spines is also involved in essential ways in noncatalytic, regulatory functions, such as dimerization, and for overall activation. The switch mechanism that converts an inactive kinase into an active kinase, for example, is thought to be embedded in the conformational changes that are correlated with assembly of the R-spine (25, 31). To test the role of the spines in LRRK2 activity and regulation, we first identified the spine residues using a structure-based alignment of all kinases in the ProKinO database (31–34). This led to a computational model of the active conformation of the kinase domain of LRRK2, where all spine residues are identified (Table 1 and *SI Appendix*, Fig. S1). For our computational modeling, we used Phyre2 (http://www.sbg.bio.ic.ac.uk/∼phyre2) and based the calculations on the protooncogene tyrosine-protein kinase SRC (35). A model based on BRAF gave similar results. Our model and the assignments of the spine residues are also consistent with the previous LRRK2 model proposed by Guaitoli et al. (36). Although we recognize that the N- and C-linker regions that flank the kinase domain are critically important for function, our calculations were restricted solely to the kinase domain (residues 1851 to 2135). Our model of the kinase domain with C and R spines assembled reflects the active conformation of the kinase domain. Most spine residues are conserved between LRRK2, PKA, and BRAF (Table 1). In particular, the C-spine residues surrounding the adenine binding pocket (CS6 to CS8) as well as the R-spine residues RS1, RS3, and RS4 are identical in PKA and LRRK2. Notably, the F185 (RS2) residue within the DFGψ motif of PKA and almost all other eukaryotic protein kinases (EPKs) is changed to a Tyr (Y2018) in LRRK2. Only a few other kinases in the human kinome (e.g., LRRK1, NEK9, and VRK1/2) have a Tyr at this position.

### Mutation of C-Spine Residues.

**Fig. 2.**
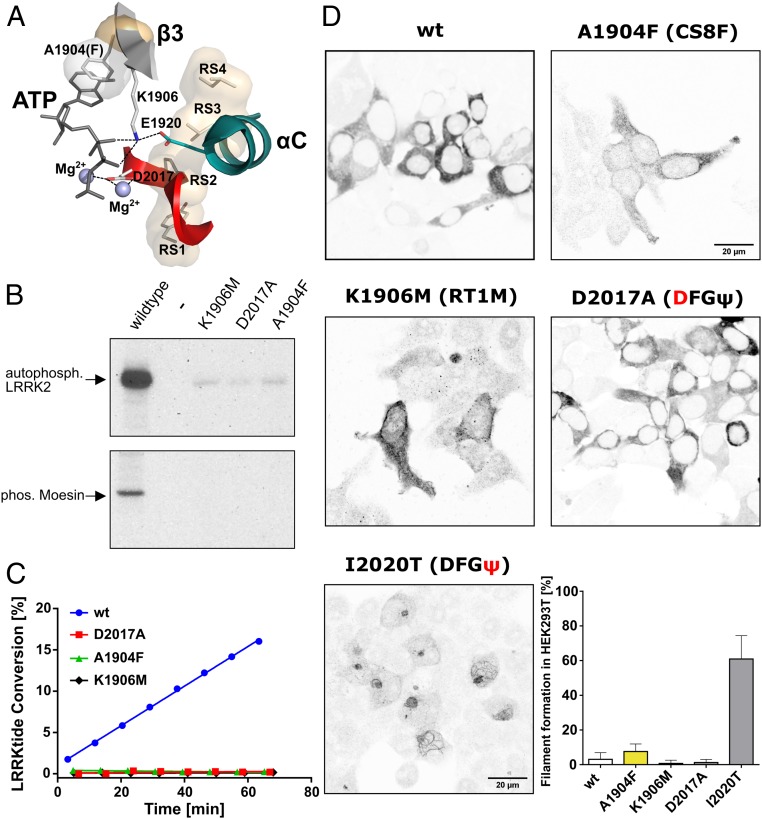
The C-spine mutation A1904F is kinetically inert. (*A*) The regulatory triad (RT1 to RT3), defined by the interaction of K1906 (RT1, β-strand 3) with E1920 (RT2, αC helix) and D2017 (RT3, DYG motif), is essential for phosphoryl transfer by promoting the correct orientation of ATP. A1904 (CS8) is critical for controlling ATP binding. Mutating any of these residues results in catalytically almost inactive kinases. (*B*) Auto- and substrate (Moesin) phosphorylation of LRRK2 wt, K1906M, D2017A, and A1904F was tested in a radioactive kinase (γ-[^32^P]ATP) assay. A1904F (CS8) shows almost no kinase activity comparable with the kinase-dead mutants, K1906M and D2017A. (*C*) Microfluidic mobility shift kinase assay with LRRKtide as a peptide substrate demonstrates no kinase activity for kinase-dead and C-spine (CS8) mutants. (*D*) A filament formation assay was performed in HEK293T cells overexpressing LRRK2 constructs 48 h after transfection. In contrast to wt, D2017A (DFGψ), and K1906M (RT1M), the C-spine mutant LRRK2 A1904F (CS8) causes filament formation in a subset of cells. The frequency of cells bearing LRRK2 filaments was drastically increased in the pathogenic mutation I2020T (DFGψ). Data are means ± SD of 2 to 5 independent experiments.

**Fig. 3.**
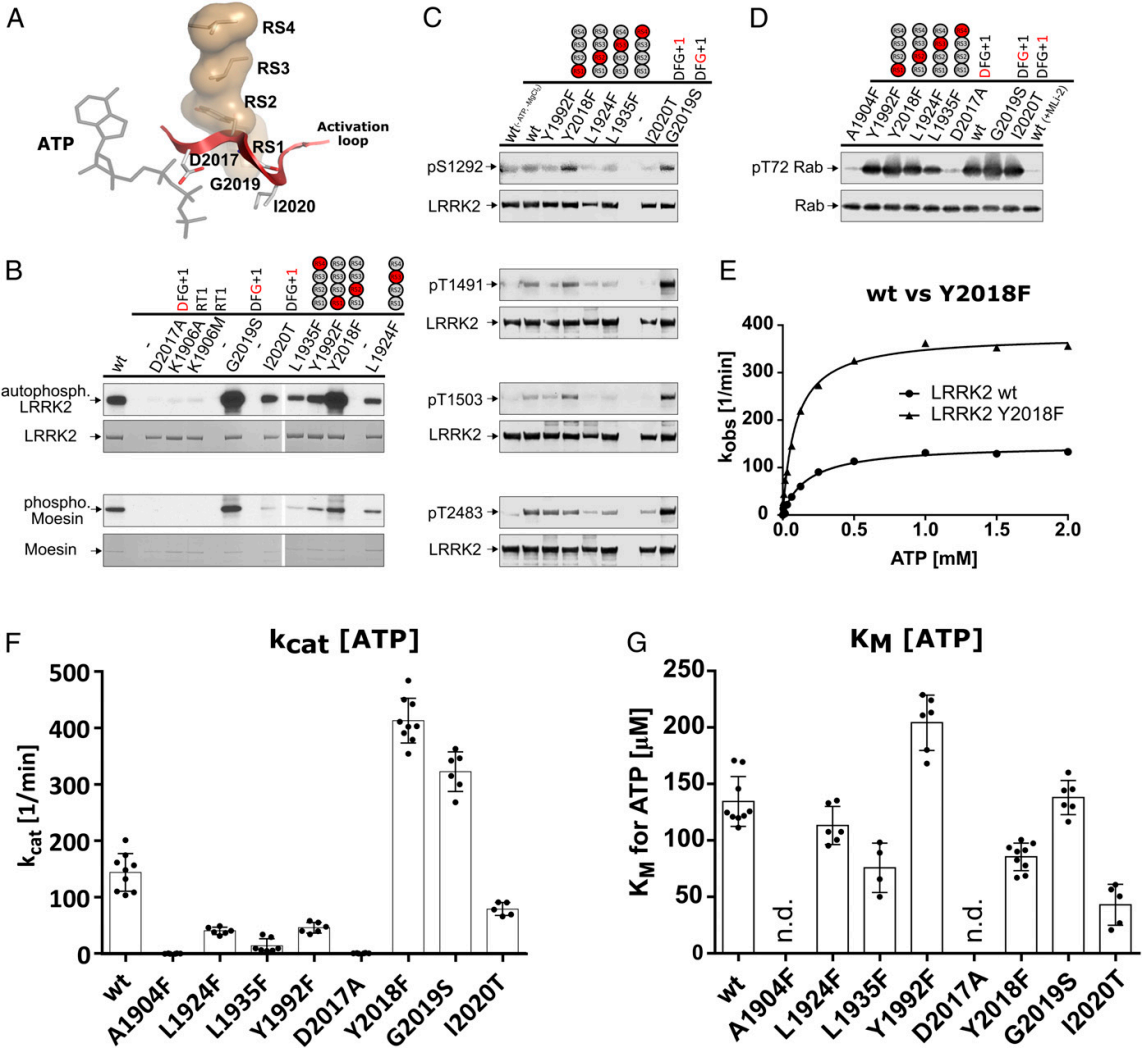
In vitro kinase assays render the LRRK2 R-spine mutation Y2018F (RS2) hyperactive. (*A*) The R-spine of LRRK2 connects 2 of the most flexible motifs in kinases, the αC helix via RS3 (L1924) and the AL (red) via RS2 (Y2018). (*B*) Based on kinase assays (Moesin) and autophosphorylation using [γ32P]-ATP, Y2018F (RS2F) was identified as a hyperactive kinase variant beside G2019S. All other R-spine mutations showed less phosphorylation. (*C*) Increased autophosphorylation was also revealed by Western blot analyses for Y2018F (RS2F) at positions S1292 and T1503 but not for T1491. G2019S, however, showed increased signals for all tested autophosphorylation sites. Y1992F (RS1F), L1924F (RS3F), and L1935F (RS4F) showed reduced autophosphorylation. (*D*) Reduced kinase activity for these mutants (Y1992F [RS1F], L1924F [RS3F], L1935F [RS4F]) was also found in an in vitro kinase assay with His-Rab8a (6-175). In contrast, Y2018F (RS2F), G2019S, and I2020T showed slightly enhanced phosphorylation of T72 in Rab8a. Rab8a phosphorylation was negligible for A1904F (CS8F), D2017A (DYG), and for the wt in the presence of 500 nM MLi-2. (*E*) Michaelis–Menten kinetics (raw data are in *SI Appendix*, Fig. S3 *C* and *D*) again demonstrate hyperactivity of Y2018F based on LRRKtide phosphorylation in a mobility shift assay. (*F* and *G*) *K*_M_(ATP) and *k*_cat_ values from Michaelis–Menten kinetics using LRRKtide as substrate. All other tested spine mutations displayed reduced kinase activity.

Having identified the spine residues in the LRRK2 kinase core, we first sought to lock the kinase into an active-like yet catalytically inert conformation based on our earlier studies with BRAF (28). By replacing the highly conserved adenine capping residue, A1904 in β3, with Phe (Fig. 2*A*), we hypothesized that the phenyl side chain would occupy the adenine binding pocket and thereby, complete the C-spine. Thus, this mutant should still assume an active-like conformation while being unable to bind ATP; this mutant would be classified as a pseudokinase. The A1904F mutation indeed abolished kinase activity using LRRKtide (RLGRDKYKTLRQIRQ) as a peptide substrate in a microfluidic mobility shift assay (Fig. 2*C*). Moesin and Rab8a phosphorylation as well as autophosphorylation were dramatically reduced (Figs. 2*B* and 3*D*). The constitutive phosphorylation of Ser910, Ser935, and Ser955 was, however, unchanged (*SI Appendix*, Fig. S2). V1893 (CS7) is another C-spine residue in the N lobe that caps the adenine ring, and mutation of V1893F also abolished kinase activity similar to A1904F and comparable with our earlier results with BRAF (*SI Appendix*, Fig. S2).

Typically, mutations of the β3-Lys (K72 in PKA, K1906 in LRRK2) in the regulatory triad (RT) (Fig. 2*A*) are done to create kinase-dead mutants. Following this line, we compared the activity of the A1904F (CS8F) mutant with K1906M (RT1M). In addition, we mutated another key residue that is critical for phosphotransfer, D2017A in the DYGψ/DFGψ motif. This also created a kinase-dead phenotype (Fig. 2 *B* and *C*). Thus, we have 3 different generic mechanisms for creating a kinase-dead mutant. K1906M (RT1A/M) contributes to positioning of the α- and β-phosphates of ATP as well as opening and closing of the active site cleft (37), D2017A controls the binding of the second metal ion and the transfer of the γ-phosphate, and A1904F sterically interferes with binding of the adenine ring.

In addition to our kinetic assays of LRRK2 catalytic functions, we utilized a cell-based assay to evaluate the capacity of LRRK2 wild-type and mutant proteins to form filaments that correlate with docking onto microtubules. We and others showed previously that 3 of the 4 common familial mutants of LRRK2 dock spontaneously onto microtubules (12, 38–42). We, therefore, transfected HEK293T cells with LRRK2 A1904F and evaluated LRRK2 localization. Overexpression of wt LRRK2 showed filament formation in only a small percentage of cells in contrast to the pathogenic mutant I2020T, which showed filaments in the majority of cells (Fig. 2*D*). Although A1904F resembles the kinase-dead mutant in terms of activity, cells expressing A1904F showed slightly more filamentous structures than cells transfected with wt LRRK2 (Fig. 2*D*). Consistently, both kinase-dead mutants D2017A and K1906M showed no filament formation. These findings suggest that the formation of spines and therefore, the conformational state of the kinase domain may also be involved in noncatalytic functions, such as cellular localization.

### The DYGψ Motif Controls R-Spine Formation and Maintains LRRK2 in an Attenuated State.

While the C-spine is essential for positioning the adenine ring of ATP and in turn, the phosphates for catalysis, the R-spine is required for assembly of an active kinase. The correlated motions of the αC helix and the DFGψ motif lead to the assembly of the conserved regulatory triad (K1906, E1920, and D2017) and in particular, the magnesium binding loop, which correlates with positioning of the γ-phosphate (Fig. 2*A*). In BRAF, the assembly of the R-spine is dynamically facilitated by phosphorylation (activation loop [AL]) and/or dimerization. To investigate the influence of R-spine assembly on kinase activity as well as cellular localization, we increased the hydrophobicity of all R-spine (RS1 to RS4) residues (Fig. 3*A*). These Phe mutants Y1992F (RS1F), Y2018F (RS2F), L1924F (RS3F), and L1935F (RS4F) were then assayed for autophosphorylation using γ-[^32^P]ATP. As shown in Fig. 3*B*, all R-spine mutants still undergo autophosphorylation; however, RS1F, RS3F, and RS4F show slightly reduced signals compared with the wt (*SI Appendix*, Fig. S7). In contrast, the DYGψ motif mutant RS2F (Y2018F) displayed increased autophosphorylation, which was also found for the hyperactive G2019S mutant. These findings were also confirmed using a phospho-specific antibody against a major autophosphorylation site, S1292 (Fig. 3*C* and *SI Appendix*, Fig. S7*A*). The same pattern (increased phosphorylation for RS2F [Y2018F] and decreased phosphorylation for RS1F, RS3F, and RS4F) was also observed for pT1491 and pT1503 (Fig. 3*C* and *SI Appendix*, Fig. S7). Interestingly, enhanced phosphorylation of pT2483 in the WD40 domain could be observed only for G2019S but not for Y2018F. Another residue that is part of the local hydrophobic environment around the DFGψ motif is F1908, which is an Leu in PKA and BRAF. Replacing L485 by Phe in BRAF created a constitutively active oncogene (26). The parallel experiment in LRRK2, replacing the corresponding Phe with Leu (F1908L), led to an ∼80% loss of activity (Table 2).

**Table 2.** Kinetic parameters of LRRK2 constructs

LRRK2 construct	*K*_M_(ATP), µM	*k*_cat_, 1/min	*k*_cat_/*K*_M_, 1/(min µM)
wt	136 ± 23	140 ± 32	1.1 ± 0.3
A1904F (CS8)	—	Inactive	—
L1924F (RS3)	113 ± 18	40 ± 6	0.36 ± 0.06
L1935F (RS4)	76 ± 22	14 ± 12	0.10 ± 0.04
Y1992F (RS1)	204 ± 25	45 ± 9	0.23 ± 0.06
D2017A (DFGψ)	—	Inactive	—
Y2018F (RS2/DYGψ)	85 ± 12	413 ± 40	4.9 ± 0.9
G2019S (DFGψ)	138 ± 15	322 ± 35	2.4 ± 0.2
I2020T (DFGψ)	43 ± 18	79 ± 12	2.3 ± 1.5
F1908L (HN1)	85 ± 9	17.4 ± 0.8	0.21 ± 0.02

Values are given as mean ± SD from at least 2 independent protein preparations with each preparation measured at least twice except for F1908L, where 1 protein preparation was measured in 4 independent experiments.

Substrate phosphorylation was next examined using a general substrate of LRRK2, Moesin, in an in vitro kinase assay. These data demonstrate again hyperactivity for Y2018F and G2019S. In contrast, all other R-spine mutations reduced Moesin phosphorylation (Fig. 3*B*). As a more physiological substrate of LRRK2, we used recombinant Rab8a. Rab8a phosphorylation was strongest for G2019S followed by I2020T and Y2018F, which still displayed a slightly higher degree of Rab8a phosphorylation compared with wt LRRK2 (Fig. 3*D* and *SI Appendix*, Fig. S7*E*). Again, these results suggest an increased kinase activity of Y2018F. In contrast to using Moesin as a substrate, the Phe mutations of RS1 and RS3 did not alter Rab8a phosphorylation, while RS4F again showed reduced activity. In vivo, Rab29 has been shown to activate LRRK2 kinase activity; so, one has to be very cautious in evaluating physiological substrates, as each substrate could also have additional mutation-independent activating or inhibiting effects (22). Therefore, it must be considered that these effects may compensate for inhibiting or activating effects of LRRK2 mutations or are even able to strengthen those.

To achieve quantitative kinetic data, we used the microfluidic mobility shift kinase assays using peptide substrate LRRKtide. For accurate determination of active kinase concentrations, we established a titration assay utilizing the high-affinity LRRK2 inhibitor MLi-2 (43). This allowed us to determine specific kinase activities independent of variations in enzyme expression and purification (*SI Appendix*, Fig. S3). We determined accurate specific activities (*k*_cat_) for all catalytically active LRRK2 mutants. Strikingly, based on the active concentration, the RS2F mutant (Y2018F) seems to be even more active than the hyperactive familial mutant G2019S (Fig. 3 *E* and *F* and Table 2).

All other R-spine mutants showed decreased yet detectable kinase activity (Fig. 3*F* and Table 2). Contrary to the Western blot assay with Rab8a, the familial kinase mutation I2020T resulted in lower kinase activity compared with LRRK2 wt. Both higher and lower kinase activities have been reported previously for I2020T depending on the assay and substrate used (1, 2, 44–49), and our analysis here points out how critical the choice of an assay can be. While small peptides allow us to quantitively determine kinetic properties, conceptionally one should appreciate that protein substrates are more physiologically relevant. We then determined the *K*_M_ values for ATP for all active mutants summarized in Table 2 (Fig. 3*G*). The *K*_M_(ATP) values for G2019S, RS3F (L1924F), and RS1F (Y1992F) were similar to wt LRRK2, while I2020T, RS4F (L1935F), and RS2F (Y2018F) all showed a 2- to 3-fold reduced *K*_M_(ATP). Having *K*_M_(ATP) and *k*_cat_ allowed us to also determine catalytic efficiency *k*_cat_/*K*_M_ (Table 2). By these criteria, the Y2018F mutant is significantly more catalytically efficient than either wt LRRK2 or G2019S (Table 2).

### Y2018F Overexpression Resembles the Phenotype of the Overexpressed Pathogenic I2020T Mutant in a Cellular Context.

**Fig. 4.**
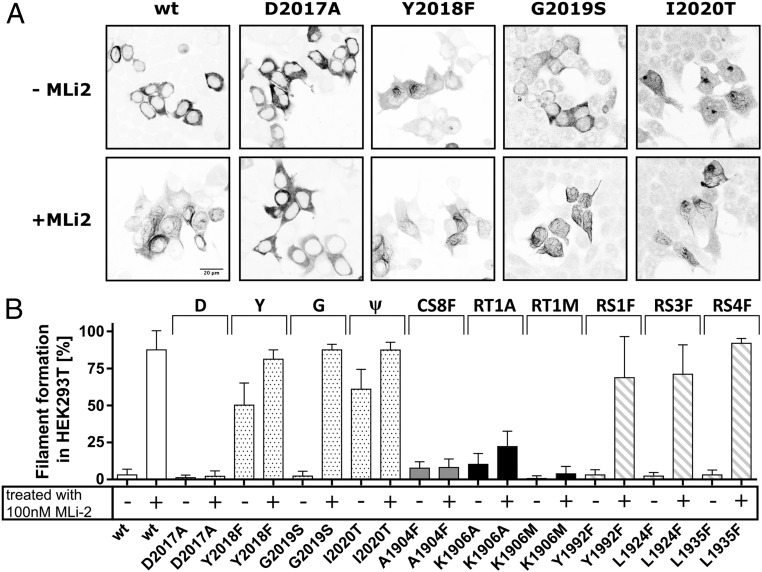
Filament formation is increased for Y2018F (RS2) and I2020T. (*A*) HEK293T cells were transfected with 1 μg of Flag-tagged LRRK2 DNA and stained with an Alexa568-tagged antibody combination against Flag. Microtubule association was defined by rod-like filamentous structures with increased fluorescence intensity, while a homogenous fluorescence distribution is considered to represent cytosolic localization of LRRK2. Representative images are shown for each variant in the absence and presence of 100 nM MLi-2. While the kinase -dead mutants (e.g., D2017A) are cytosolically distributed in the presence of MLi-2, most constructs display filament formation within 60 to 80% of the transfected cells. In the absence of inhibitor, all LRRK2 constructs are cytosolically distributed except Y2018F and I2020T, which show a filamentous pattern of expression in 50 to 60% of the transfected cells. (*B*) Percentages of cells exhibiting LRRK2 filament formation. Data are means ± SD of 2 to 5 independent experiments. Each transfected construct was measured at least twice in the absence (−) and in the presence (+) of 100 nM MLi-2.

All R-spine mutants were probed in a cellular context for filament formation. HEK293T cells were transiently transfected with the plasmids coding for the respective full-length proteins and treated with MLi-2 or dimethyl sulfoxide (DMSO) (control). In line with previously reported results, wt LRRK2 as well as G2019S showed a cytosolic distribution in the absence of the inhibitor (12). Treatment with a specific LRRK2 inhibitor for 2 h (MLi-2, 100 nM) induced filament formation of the wt and G2019S in the majority of cells (approximately 80%) (Fig. 4).

Strikingly, the DYGψ motif mutant RS2F (Y2018F) showed filament formation even in the absence of MLi-2, and treatment with MLi-2, similar to I2020T, further enhanced the proportion of cells exhibiting microtubule association (Fig. 4). Thus, the conformation and/or flexibility of the Y2018F and I2020T mutants in the absence of inhibitor are somehow different from wt LRRK2 and G2019S. The Y2018F mutant also demonstrates that filament formation does not correlate with increased kinase activity. All other R-spine mutations showed robust filament formation only in the presence of MLi-2. These results reinforce the hypothesis that filament formation and microtubule association are conformation-driven effects.

We also tested the kinase-dead mutants described above to see if MLi-2 induced filament formation. The 2 kinase-dead mutations K1906M (RT1M) and D2017A (DFGψ/RT3) consistently showed no filaments independent of inhibitor treatment (Fig. 4 and *SI Appendix*, Fig. S4). Filament formation associated with the C-spine mutant (A1904F) was also not enhanced by binding of MLi-2, consistent with our hypothesis that the ATP site is sterically blocked. Interestingly, the K1906A mutant showed a slight increase in filament formation in the presence of MLi-2, suggesting that the inhibitor can still bind to the nucleotide site, even though the protein lacks catalytic activity. These phenotypic differences in the ability of different mutants, including the pathological mutants, to induce filament formation and association with microtubules may provide important insights for the design of kinase inhibitors that do not promote this filamentous pattern.

### PKA Model.

To ask whether introducing a tyrosine into the DFGψ motif in other kinases may lead to inhibition, we used the PKA catalytic subunit as a model. Specifically, we mutated F185 (Y2018 in LRRK2) to Tyr. In addition, we changed G186 (G2019 in LRRK2) to Ser and also, generated the double mutant (F185Y/G186S). We expressed, purified, and assayed the resulting mutants using the peptide substrate PKStide (PKIα [14–22] A21S, GRTGRRNSI-NH_2_). As seen in *SI Appendix*, Fig. S5, the mutation F185Y did not have an inhibitory effect on the C subunit as for LRRK2; instead, it was significantly more active than the wt C subunit. The other 2 mutants also show an increase in kinase activity. The results emphasize that the switch mechanism is highly specific for each protein kinase, and each kinase is sensitive to subtle changes in the DFGψ motif.

## Discussion

Protein kinases have evolved to be highly regulated molecular switches that are precisely activated in response to specific biological cues. In LRRK2, the kinase domain is embedded in a large multidomain protein, and much is still unknown about the biological role of LRRK2 and how it is activated. Using a combination of structure-based analysis and site-directed mutagenesis accompanied with cell-based microtubule decoration assays, we discovered a previously unappreciated regulatory mechanism for the LRRK2 kinase domain. Based on our studies of BRAF, a close LRRK2 homolog, we hypothesized that the dynamic switch mechanism for activation of LRRK2 would be embedded in the kinase domain, specifically in the conformational changes that lead to the assembly of the hydrophobic R-spine (50). By exploring the role of the R-spine residues and more specifically, the DYGψ motif, we discovered that the DYGψ tyrosine is a key residue for controlling the switch mechanism. Replacing Y2018 with Phe releases the brake that holds the inactive conformation in place and leads to the creation of a hyperactive kinase. In addition, we explored 3 different mechanisms that create kinase-dead pseudokinases, which further emphasize the synergistic role of hydrophobic and hydrophilic/charged residues in the activation of LRRK2 and of all active kinases.

The importance of the DFGψ motif and the R-spine is further emphasized by the fact that 2 of the PD risk factor mutations in LRRK2, G2019S, and I2020T are localized within this motif. This information plus our analysis allow us to better appreciate the detailed features of this finely tuned mechanism. The principles also build on our previous analysis of BRAF, where we initially validated the importance of the spine hypotheses (26). The DFGψ motif is indeed a “hot spot” for mutations in cancer-associated kinase oncogenes (30), and therefore, we anticipate that there will be many other examples where simply releasing the brake (in this case, a simple hydroxyl group) may be sufficient to create an oncogene.

**Fig. 5.**
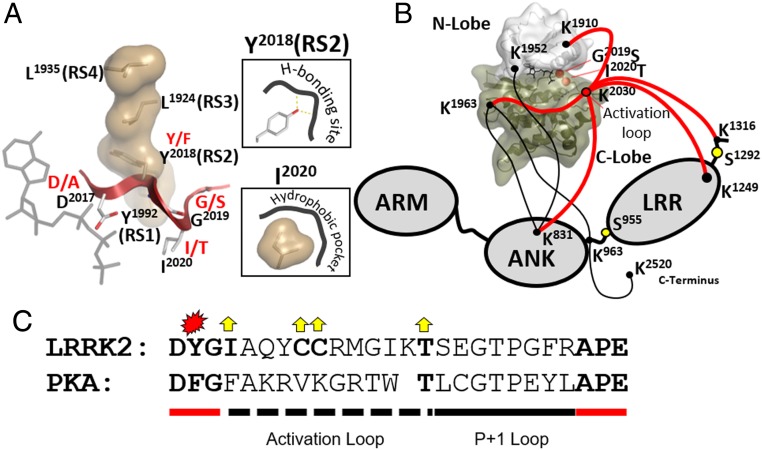
The kinase domain of LRRK2 is a critical hub for internal domain organization with an essential role of the R-spine to control function and activity. (*A*) The assembly of the R-spine needs to be tightly regulated to control the conformation of the DYGψ motif and thereby, the ON and OFF states of the kinase. Pathogenic mutations in this motif (i.e., G2019S [DYGψ] and I2020T [DYGψ]) alter kinase regulation and become a driving force for PD. With Y2018F (RS2/DYGψ), we describe a mutation resembling features of both pathogenic mutations (i.e., hyperactive as G2019S and prone to form filamentous structures, like I2020T). Both effects seem to be promoted by an active kinase conformation. For Y2018F, this might be driven by a loss of hydrophilic interactions in the DYG-out conformation, while in the case of G2019S, the Ser reduces the flexibility of the Mg-positioning loop and stabilizes the ON state. For I2020T, we hypothesize that a hydrophobic interaction in the DYG-out conformation with a not yet defined hydrophobic pocket is lost. (*B*) Cross-linking experiments (cross-links are shown as red and black lines) by Guaitoli et al. (36) revealed several links of the kinase domain with other domains of LRRK2, such as the ANK and LRR domain. Especially K2030 (red lines) and thereby, the AL seem to be localized to important domain interfaces in full-length LRRK2 as well as key phosphorylation sites (yellow dots). (*C*) Sequence comparison of the AL and the P + 1 loop of LRRK2 and PKA. The AL harbors 2 adjacent cysteines, which may be important for oxidation sensing.

To appreciate the implications of the Y2018F mutation, it is useful to look more closely at the integration of the R-spine and the DFGψ motif. The R-spine is composed of 4 residues, and each is linked to a conserved functional motif (Fig. 1*E*). RS1 is firmly anchored to the catalytic loop in the C lobe, while RS4 is anchored to β4 in the N lobe. In contrast, the intervening R-spine residues RS2, embedded in the DFGψ motif, and RS3, anchored to the αC helix, are highly dynamic. The correlated motions of the DFGψ motif and the αC helix lead to the assembly of the essential regulatory triad at the active site cleft (Fig. 2*A*), which allows for the precise orientation of the γ-phosphate so that it is poised for transfer to a protein substrate (25). Each residue in the DFGψ has a specific role to play. The Asp (D2017) is the residue that must be precisely positioned at the active site to allow for the transfer of the γ-phosphate (Fig. 5*A*). In most kinases, the second residue is a Phe, which provides a flexible hydrophobic driving force or “grease” for the assembly of the R-spine. While Y2018 in LRRK2 also provides hydrophobicity, there is an important difference. It also has a hydroxyl moiety, which can provide additional interactions that precisely regulate the positioning of the side chain. Replacing the Y2018 with Phe retains the hydrophobicity but abolishes the precise positioning that is mediated by the hydroxyl moiety, and this leads to an unleashed kinase. A stabilized “OFF” state for Y2018 presumably favors a disassembled, inactive R-spine conformation (Fig. 5*A*), and identifying the pocket that provides the hydrogen bond donor and/or acceptor will be an important future challenge. G2019S, the most common risk factor mutation, is also hyperactive but most likely for different reasons. The conserved glycine in this motif is almost certainly there to provide a highly flexible hinge to mediate the transition from active to inactive. The equilibrium in most kinases will favor the inactive conformation. The transition from active to inactive is slowed down, and in LRRK2, the simple replacement of the Gly with Ser favors the active conformation (51). In most kinases, the ψ residue, I2020 in LRRK2, is hydrophobic. This residue has a role in shielding the active site from water in the active conformation and may also contribute to recognition of the P + 1 residue in the protein/peptide substrate (52). We predict that there is also a hydrophobic pocket in full-length LRRK2 that contributes to stabilization of the OFF state (Fig. 5*A*). A structure of full-length LRRK2 will be needed to identify this pocket.

Enhancing the hydrophobicity of the spine residues and their neighboring environment is another strategy that was exemplified by the BRAF mutants (27, 28). In the case of LRRK2, we find that increasing the hydrophobicity of the other spine residues actually led to a reduction in activity, although we did not further explore the mechanism. We emphasize that the hydrophobic space of each kinase must be carefully examined. Most of the hydrophobic residues in the R-spine and the surrounding residues are conserved in PKA, BRAF, and LRRK2 with 1 exception. Residue 1908 in β3 is a Phe in LRRK2 but an Leu in PKA in BRAF. Mutating this residue to Phe in BRAF is an oncogenic mutation that enhances the hydrophobic contacts between β3 and the αC helix (26). This is consistent with our finding that approximately 80% of the LRRK2 kinase activity is lost when F1908 is replaced with Leu. We suggest that F1908 may contribute to the strong activating phenotype of the Y2018F mutant. After the brake mechanism associated with the Tyr hydroxyl moiety is broken, LRRK2 is poised to be stabilized in an active conformation. In general, the hydrophobic environment around the spine residues in LRRK2 is more extensive than in PKA and BRAF, which may also contribute to the strong activating phenotype of Y2018F. Once again, we emphasize that the hydrophobic space of the core is kinase specific; each kinase must be analyzed individually.

### Assembly of the Regulatory Triad.

The assembly of the hydrophilic regulatory triad at the active site cleft is a direct and synergistic consequence of the hydrophobic-driven assembly of the R-spine (25). What do these 3 residues do, and why are they so important? The Lys (K1906 in LRRK2) is anchored to the β-sheet, which is rigid. It provides a “lighthouse beacon” to guide the positioning of the Glu (E1920 in LRRK2) in the αC helix and anchors the α- and β-phosphates of ATP. The Glu integrates the αC helix with the β-sheet, creating a functional N lobe that is anchored to the AL (53). This pair also coordinates opening and closing of the cleft during the catalytic cycle (37). The Asp (D2016 in LRRK2) in the DFGψ motif binds to the second metal ion and the γ-phosphate of ATP. This Mg ion is the critical “linchpin” that is essential for catalysis and for release of ADP (54, 55). Most kinase crystal structures do not have an ordered γ-phosphate and do not have the second metal ion. Here, we confirm the inactivating consequences of K1906 and D2017 mutations. Our biggest challenge now is to elucidate the inactive conformation of wt full-length LRRK2 where the regulatory triad is broken. As seen in *SI Appendix*, Fig. S6, we have no template for this, and the conformation of most inactive kinases is not known, because the adjacent regulatory domains are deleted.

### Activation Loops.

We and others have modeled the active conformation of LRRK2 based on the structure of SRC and many other kinases. BRAF is a good model for comparison, as LRRK2 belongs to the same branch of the kinome tree. Although much smaller, BRAF also provides a good model for how an N-terminal domain (NTD) can reach over and stabilize the inactive state, even though we do not yet have a structure of full-length BRAF. As with most kinases, the terminal inhibitory domain of BRAF is typically cleaved off to obtain a crystal structure of the kinase domain bound to inhibitors. The NTD in BRAF is predicted to reach over and cover the active site cleft precisely in the region where the N and C lobes come together. This is the home of the AL, and while we have many structures now of ALs in active or semiactive conformations, in most inactive kinases the AL is disordered, suggesting that ordering is imposed by domains and/or motifs that lie outside the kinase domain (*SI Appendix*, Fig. S6). There are a few cases, such as SRC, where the AL is ordered very differently in the inactive state, and here, we can see how the same residues that are essential for the active conformation can play different roles in stabilizing the inactive conformation. The E91^PKA^ (RT2) equivalent in the αC of inactive SRC, for example, is interacting with the DFGψ + 2 Arg that will be part of the AL network when the AL-Tyr is phosphorylated. In LRRK2, we predict that this residue will be contributing to the active conformation of the AL as it does in every other protein kinase. However, we also predict that this region will likely be contributing to stabilizing the inactive conformation of full-length LRRK2. Specifically, based on cross-linking of the full-length inactive LRRK2 (36), K2030 in the AL is a key residue for cross-linking to different parts of the protein (Fig. 5*B*). Two major links are to the LRR domain (K1249 and K1316), and 1 is to the Ankyrin repeats (K831). There are also 2 cross-links within the kinase domain (K1910 and K2065). While the details of these cross-links need to be validated, they nevertheless suggest that the AL loop will be localized to important domain interfaces in the full-length kinase that will likely include the LRR and ankyrin repeats. This further supports the model that the N-terminal region of LRRK2 will be shielding the active site of the kinase domain in full-length LRRK2 as we have already proposed earlier (13). Two adjacent Cys residues are another intriguing feature of the LRRK2 AL, and these might be sensitive to oxidation (Fig. 5*C*). Other than providing hypotheses for the development of potent inhibitors, identifying the docking sites for Y2018 and I2020 is an important future challenge to further understand the mechanism of LRRK2 activation as well as how pathogenic mutations hijack this process. Our analysis of LRRK2 also provides a general strategy for analyzing the conformational dynamics of any unknown protein kinase, even very large ones such as LRRK2.

## Methods

### Cell Culture and Transfection.

LRRK2 wt and mutants were expressed with an N-terminal Flag-Strep-Strep tag in HEK293T cells. For this, 1.0 × 10^7^ cells were seeded on 15-cm Ø cell culture dishes followed by a 24-h incubation at 37 °C and 5% CO_2_ in Dulbecco’s modified eagle medium (DMEM) high glucose (with l-glutamine; without sodium pyruvate; Biowest) supplemented with 10% fetal bovine serum (FBS). Subsequently, cells were transfected with 15 μg per dish plasmid DNA (pCDNA3.0-FSS-LRRK2; NM_198578), which was incubated for 30 min with 150 μL per dish polyethylenimine in 1.5 mL DMEM high glucose and then added to each dish. After 24 h, the medium was exchanged with fresh DMEM (high glucose, +10% FBS), and cells were incubated for another 24 h before harvesting. Cell pellets were stored at −20 °C before use.

### Purification of Flag-Strep-Strep–Tagged LRRK2 Constructs.

Cell pellets were lysed in fresh, ice cold lysis buffer (25 mM Tris⋅HCl, pH 7.5, 150 mM NaCl, 10 mM MgCl_2_, 0.5% Tween 20, 500 µM guanosine diphosphate (GDP), cOmplete ethylenediaminetetraacetic acid (EDTA) free protease inhibitor mixture [Roche], PhosSTOP [Roche]) followed by a 30-min incubation at 4 °C. Cell debris was removed by centrifugation at 42,000 × *g* and 4 °C for 40 min. After filtration (0.45-µm sterile filter), the supernatant was transferred on a Streptactin Superflow column (0.5 mL bed volume; IBA Goettingen). Streptactin affinity purification was in principle performed according to the manufacturer’s protocol, except that all buffers were supplemented with 500 µM GDP (Biolog Life Science Institute) and 10 mM MgCl_2_. Eluted protein LRRK2 was supplemented with 10% glycerol and 0.5 mM Tris(2-carboxyethyl)phosphine (TCEP) and stored at −80 °C. Concentrations were determined using the Bradford Protein Assay (56). Buffers used for LRRK2 purifications were used for autophosphorylation and Moesin phosphorylation assays, except that no additional MgCl_2_ was added.

### Radioactive Phosphorylation Assays.

Autophosphorylation of LRRK2 variants was analyzed using [γ^32^P]-ATP. For this, Kinase Buffer (Cell Signaling) was supplemented with 500 μM guanosine triphosphate (GTP), 100 µM ATP, and 17 nM (≡1 µCi/37 kBq) [γ^32^P]-ATP. Each reaction was started by addition of 200 ng LRRK2 (wt or mutant). The reaction mix was incubated for 1.5 h at 30 °C. The reaction was stopped by addition of NuPAGE sample buffer (Thermo Fisher Scientific) and by incubation at 95 °C for 2 min. Samples were separated using a NuPAGE Gel (4 to 12%) and stained with Coomassie brilliant blue G for documentation. The gel was then incubated in a solution of 5% glycerol and 20% EtOH followed by an overnight drying step while pinched between 2 layers of cellophane. Radioactive autophosphorylation was detected by photo film (24-h exposure at −80 °C).

Substrate phosphorylation was analyzed similarly, except that the reaction was supplemented with 480 μg/mL GST-Moesin.

### Autophosphorylation Analysis with Phospho-Specific Antibodies.

LRRK2 spine variants were tested for (auto-)phosphorylation with phospho-specific antibodies from rabbit: pS910 (Epitomics #5098–1), pS935 (Epitomics #5099–1), pS955 (Abcam MJF-R11 [75-1]), pS1292 (Abcam MJF 19–7-8), pT1491 (Abcam MJF-R5 [88-3]), pT1503 (Abcam MJF-R6 [227-1a]), and pT2483 (Abcam MJF-R8 [21-2e]). For this, 200 ng LRRK2 was incubated in Kinase Buffer (Cell Signaling) supplemented with 500 μM GTP and 100 µM ATP for 1.5 h at 30 °C. After sodium dodecyl sulfate polyacrylamide gel electrophoresis (SDS/PAGE) and blotting, the respective nitrocellulose membrane was blocked with TBS-T (50 mM Tris, pH 7.4, 150 mM NaCl, 0.1% [vol/vol] Tween-20) containing 5% (wt/vol) bovine serum albumin (BSA) and was subsequently incubated overnight with primary antibodies diluted in TBS-T (1:1,000). Total LRRK2 was detected using an anti-Flag antibody (1:1,000, clone M2, mouse; Sigma-Aldrich). Fluorescently labeled secondary antibodies (anti-rabbit IRDye800CW and anti-mouse IRDye680RD, 1:15,000; LiCOR) were used for the parallel detection of phospho-specific and anti-Flag using an Odyssey FC imaging system (LiCOR).

### Western Blot of Rab8a Phosphorylation by LRRK2 Spine Mutations and Pathogenic Variants.

Rab8a phosphorylation by LRRK2 was tested using a phospho-specific antibody against phospho-Thr72 of Rab8a. Therefore, kinase assays containing 5 µM (6xHis)-Rab8a (6-175), 100 nM respective LRRK2 mutant, 25 mM Tris⋅HCl, pH 7.5, 50 mM NaCl, 10 mM MgCl_2_, 1 mM ATP, 0.5 mM GTP, 0.1 mg/mL BSA, and 1 mM dithiothreitol (DTT) were performed at 30 °C for 30 min. Reactions were stopped by adding sample buffer and denaturing the samples at 105 °C for 5 min. Samples were separated via SDS/PAGE and blotted onto a nitrocellulose membrane. Subsequently, the membrane was blocked with 5% (wt/vol) BSA in TBS-T for 1 h. Primary antibody incubation was performed overnight at 4 °C with the phospho-specific anti-Rab8a (phospho-T72) antibody (MJF-R20, rabbit; abcam) and an anti-His antibody (mouse; GE Healthcare). After secondary antibody incubation (anti-rabbit IRDye800 and anti-mouse IRDye680, 1:15,000; LiCOR) for 1 h at room temperature, signals were detected with the Odyssey FC imaging system (LiCOR). FSS-LRRK2 wt and mutants were detected on an additional blot using an anti-Flag antibody (1:1,000, clone M2, mouse; Sigma-Aldrich) in combination with an anti-mouse antibody (anti-mouse IRDye680RD, 1:15,000; LiCOR).

### Microfluidic Mobility Shift Kinase Assay.

Michaelis–Menten kinetics of LRRK2 for ATP were determined via a microfluidic mobility shift assay combined with kinase assays using LRRKtide (RLGRDKYKTLRQIRQ-amide; GeneCust) as a substrate. Microfluidic mobility shift kinase assays were performed using assay buffer (25 mM Tris⋅HCl, 50 mM NaCl, 1 mM DTT, 0.5 mg/mL BSA) containing 950 µM LRRKtide and 50 µM fluorescein-LRRKtide (LRRKtide solution), 50 nM LRRK2, 10 mM MgCl_2_, 0.5 mM GTP, and the respective ATP dilution. Each reaction was started by adding the LRRKtide solution to 384-well plates. A LabChip EZ Reader (PerkinElmer, Inc.) was used to monitor substrate conversion for 1 h at 30 °C. The percental conversion was plotted against time, and the slope (*m* = percentage per minute) was determined using a linear fit. Subsequently, the slopes were converted into reaction velocities (*v*_0_ = micromoles per minute), and plotted against the respective ATP concentration, and *K*_M_ as well as *v*_max_ values were determined using a Michaelis–Menten fit. Measurements were performed in triplicates with at least 2 independent protein preparations.

The kinase assay was also used to determine active protein concentrations by titrating each LRRK2 preparation with the high-affinity inhibitor MLi-2 (Merck). Therefore, 24 µL of Buffer A (25 mM Tris⋅HCl, 50 mM NaCl, 1 mM DTT, 0.5 mg/mL BSA, 104.2 nM LRRK2) was mixed with 1 µL of an MLi-2 dilution series (50× concentrated), which was prepared in 100% DMSO. Subsequently, 10 µL of this reaction mix was added to 10 µL of Buffer B (25 mM Tris⋅HCl, 50 mM NaCl, 1 mM DTT, 0.5 mg/mL BSA, 1,900 µM LRRKtide, 100 µM fluorescein-LRRKtide) containing *x* µM ATP (*x* = *K*_M_[ATP] of the respective LRRK2 mutant). Substrate conversion was monitored as described above, and resulting conversion rates were plotted against the respective MLi-2 concentrations. Active protein concentrations were obtained by determining a linear fit (assuming an 1:1 binding of MLi-2).

### Immunofluorescence and Laser Confocal Imaging.

HEK293T cells were seeded onto 6-well dishes containing poly-d-lysine–coated glass coverslips or onto poly-d-lysine–coated glass-bottom dishes (MatTek Corporation). Cells were transfected with the Lipofectamine 2000 reagent (ThermoFisher Scientific) per the manufacturer’s protocol using 1 μg of tagged LRRK2 DNA. Transfected cells grew for 48 h. They were then treated with the LRRK2 inhibitor MLi-2 for 2 h before fixation with 4% paraformaldehyde in phosphate-buffered saline (PBS) for 15 min at room temperature. Cells were washed in PBS, permeabilized in 0.1% Triton X-100, and blocked in 1% BSA, 50 mM glycine, and 2% normal serum. The primary rabbit anti-Flag antibody (catalog no. PAB0900; Abnova Company) was mixed at a dilution of 1:250 in blocking buffer diluted 5-fold in PBS and applied for 1 h at room temperature. The secondary antibody (donkey anti-rabbit Alexa568, catalog no. A10042; Invitrogen) was diluted (1:100) in the same buffer and applied for 1 h at room temperature. Samples were mounted with the antifade agent ProLong Gold with 4′,6-diamidino-2-phenylindole (DAPI) (ThermoFisher Scientific). Confocal imaging was performed with the Olympus Fluoview 1000 laser scanning confocal microscope using a 60× oil immersion objective lens with numerical aperture 1.42. *Z*-stack images were acquired with a step size of 0.3 μm and processed using the Fiji software package (57). Cells expressing the different mutants were assessed for the presence of clear filamentous structures and quantified in 2 to 5 independent experiments.

## Supplementary Material

Supplementary File
