# Supplementary material for: The dynamic switch mechanism that leads to activation of LRRK2 is embedded in the DFGψ motif in the kinase domain
Source: Proc Natl Acad Sci U S A. 2019 Jul 10;116(30):14979–88. doi: 10.1073/pnas.1900289116 (PMC6660771; doi:10.1073/pnas.1900289116)
Supplement: Supplementary File [file pnas.1900289116.sapp.pdf]

Supporting Information Appendix

# The dynamic switch mechanism that leads to activation of LRRK2 is embedded in the DFGψ motif in the kinase domain

Sven H. Schmidt[a], Matthias J. Knape[a], Daniela Boassa[b,c], Natascha Mumdey[a], Alexandr P. Kornev[d], Mark H. Ellisman[b,c], Susan S. Taylor[d1], and Friedrich W. Herberg[a1]

[a]Department of Biochemistry, University of Kassel, 34132 Kassel, Germany;

[b]National Center for Microscopy and Imaging Research, University of California, San Diego, La Jolla, CA 92093, USA;

[c]Department of Neurosciences, University of California, San Diego, La Jolla, CA 92093, USA;

[d]Department of Pharmacology, University of California, San Diego, La Jolla, California 92093, USA

1 Corresponding author

www.pnas.org/cgi/doi/10.1073/pnas.1900289116

# Abbreviations:

ADP, adenosine diphosphate; AL, activation loop; ATP, adenosine triphosphate; BSA, bovine serum albumin; BRAF, Serine/threonine-protein kinase B-raf; CS, Catalytic Spine; DMEM, Dulbecco's Modified Eagle Medium; DMSO, dimethylsulfoxide; FBS, fetal bovine serum; fl, full length; GTP, guanosine triphosphate; GST, Glutathione-S-Transferase; HEK293T, human embryonic kidney cells 293T

# Supplementary Figures:

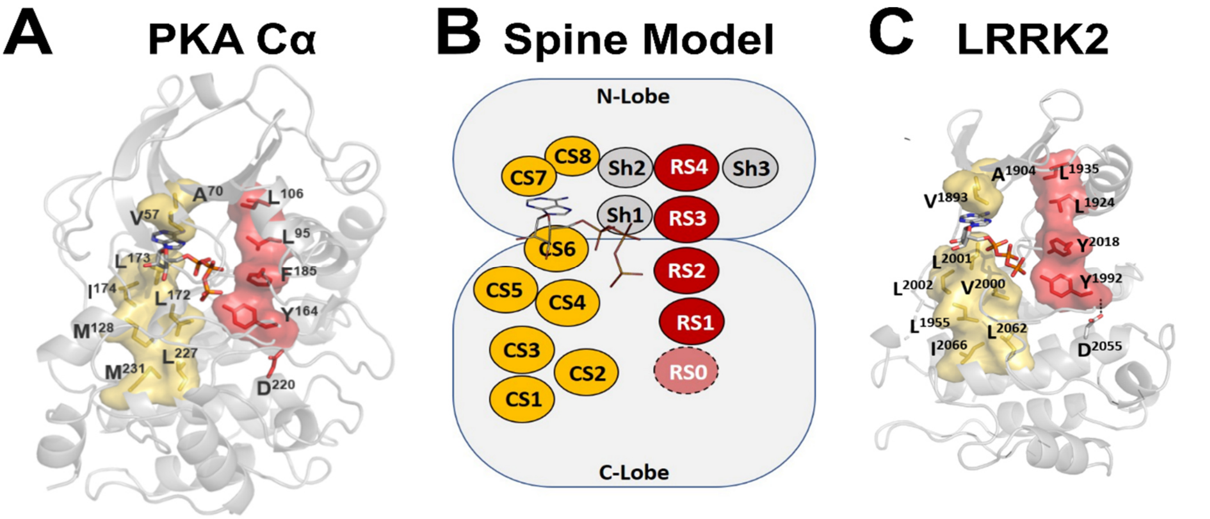


**Figure S1:** The internal architecture of a protein kinase is defined by two hydrophobic motifs. These "spines" need to be assembled in order to generate an active kinase. The C-spine (yellow) is composed of 8 residues (CS1-CS8) whereas the R-spine (red) consists of 4 residues (RS1-RS4). The shell residues (Sh1-Sh3, gray) in the N-lobe link the R-spine with the C-spine.

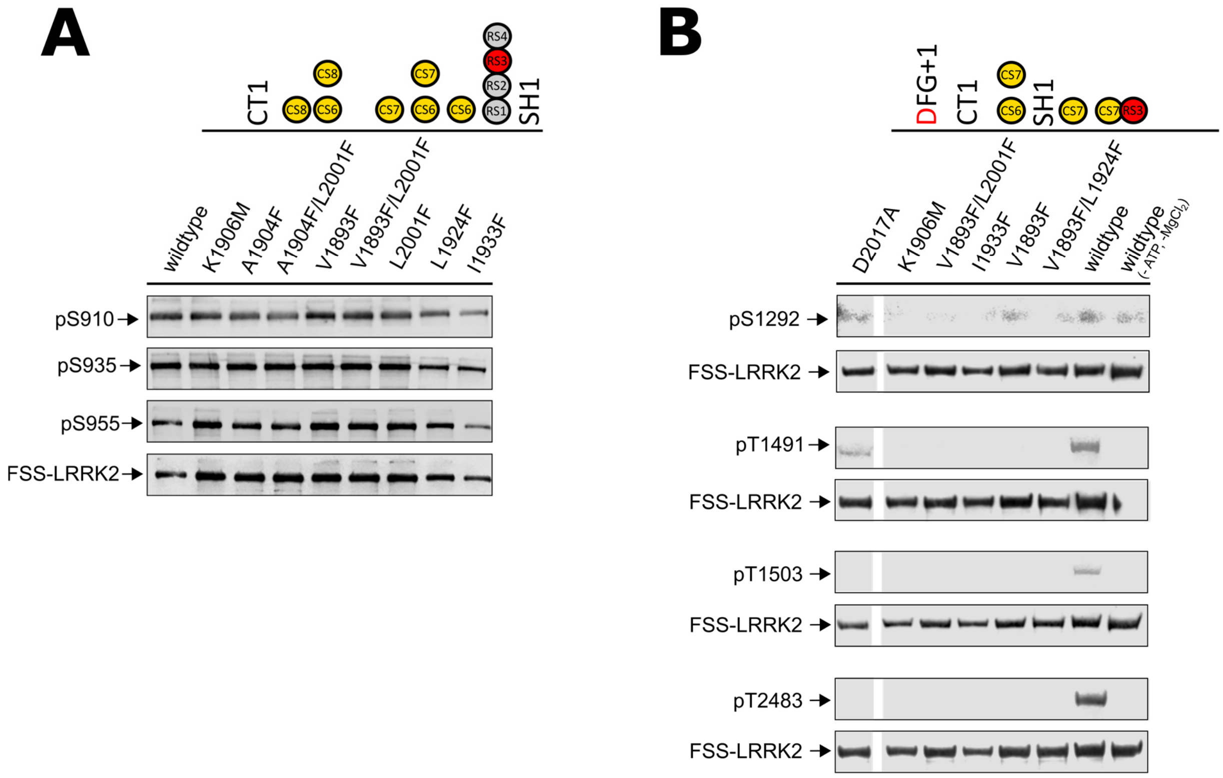


**Figure S2: Autophosphorylation of LRRK2 spine mutants.** Autophosphorylation of LRRK2 mutants was tested in an in vitro kinase assay by incubating the respective kinase in buffer with ATP. LRRK2 phosphosite specific antibodies (α-pS910, α-pS935, α-pS955, α-pS1292, α-pT1491, α-pT1503, α-pT2483; dilution 1:1000) were used for detection. **(A)** The phosphorylation cluster between ANK and LRR domain is not affected by LRRK2 spine mutations or kinase dead mutations (e.g. K1906M [RT1M]). **(B)** Phosphorylation pattern of potential autophosphorylation sites of selected LRRK2 spine and shell mutants as well as a kinase dead control (K1906M). All tested mutants showed no or weak autophosphorylation.

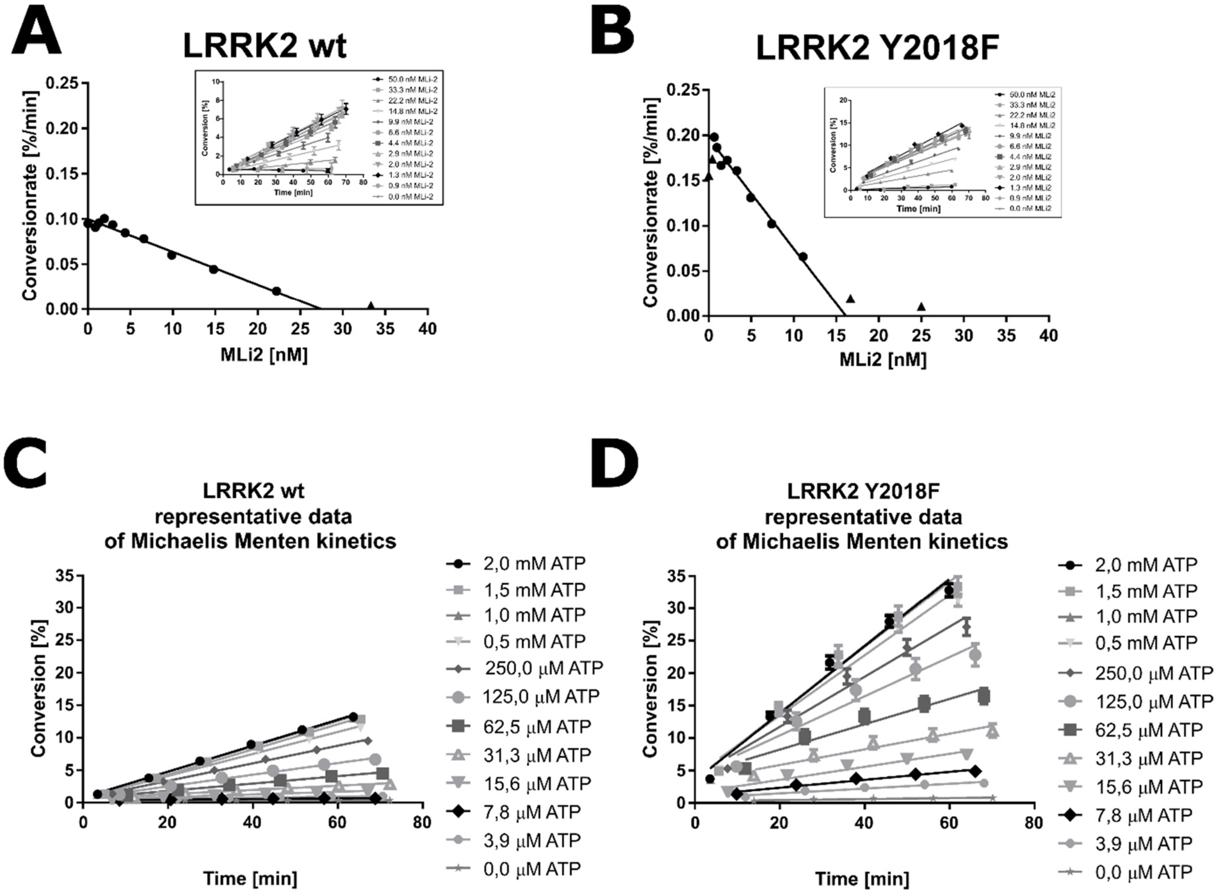


**Figure S3: Determination of active kinase concentrations.** Representative active concentration determinations are shown for LRRK2 wt **(A)** and Y2018F (RS2) **(B)** using a novel inhibitor-(MLi-2)-titration based kinase assay. 50 nM [as determined with the Bradford assay] of each LRRK2 variant were used in the presence of varying MLi-2 concentrations. The percental conversion of Fluo-LRRKtide for each reaction was recorded over time in a microfluidic mobility shift assay (s. inset). The resulting conversion rates (slope for each reaction) were plotted against the respective MLi-2 concentrations. Based on a 1:1 binding stoichiometry of the inhibitor to the kinase, the X-intercept equals the active LRRK2 concentration. **(C, D)** Raw data of Michaelis Menten kinetics of LRRK2 wt and Y2018F.

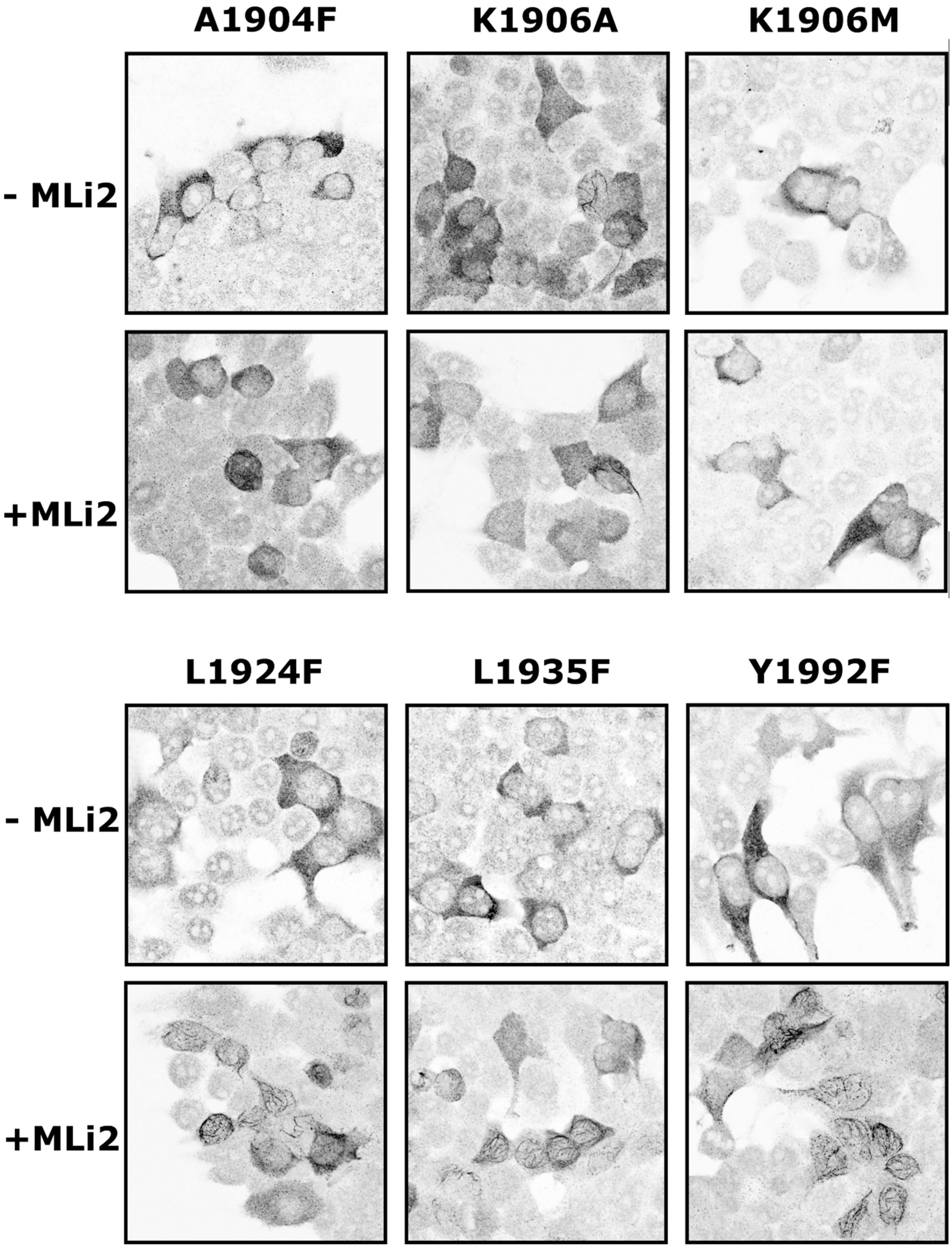


**Figure S4: LRRK2 R-spine mutants form filaments in cells upon treatment with the inhibitor MLi-2.** HEK293T cells were transiently transfected with Flag-tagged LRRK2 mutants. Cells were either treated with 100 nM MLi-2 or DMSO (negative control). Only the R-spine mutations led to a robust microtubule association under MLi-2 treatment, while cells expressing kinase dead mutants showed little to no filamentous structures independent of the treatment. Nevertheless, filament formation was slightly increased in A1904F and K1906A compared to wt or R-spine mutations in the absence of the drug.

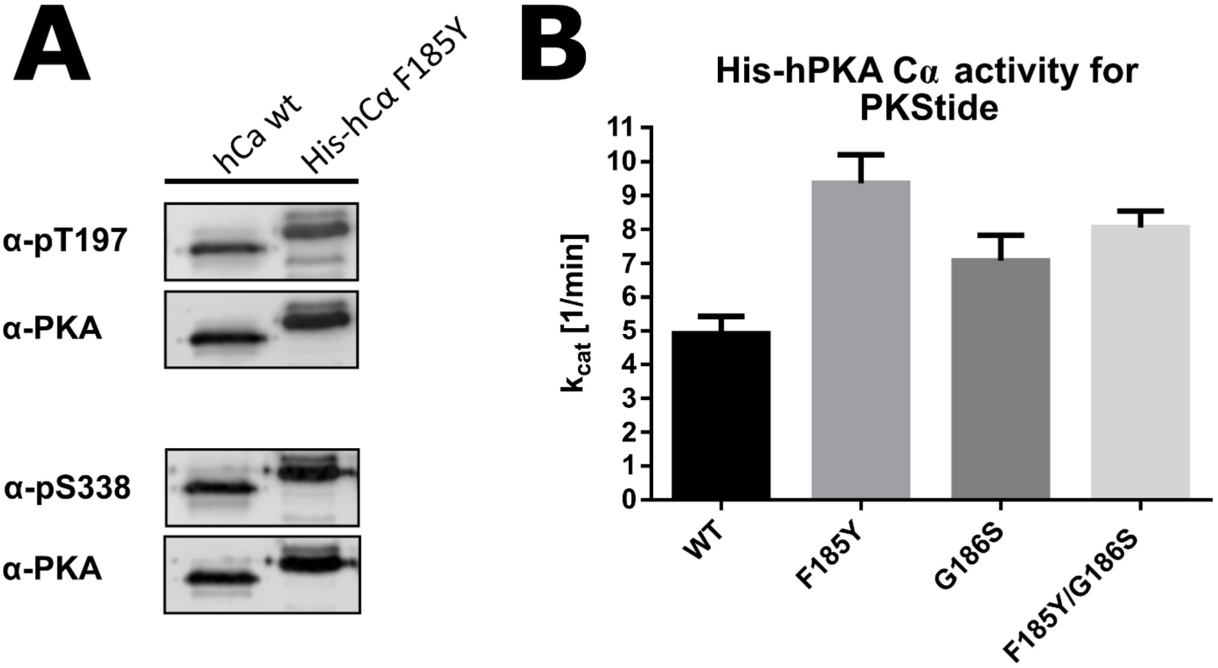


**Figure S5: Introducing a DYG motif into PKA. A)** Western blot showing the autophosphorylation state for Thr197 (AL) and Ser338 (C-tail) of His-hPKA Cα wt and His-hPKA Cα F185Y. **B)** Kinase activities of His-hPKA Cα wt and various DFGψ mutations: G186S (G2019S in LRRK2), F185Y (wt DYG motif in LRRK2). In contrast to LRRK2 a DYG instead of a DFG motif increases kinase activity of PKA.

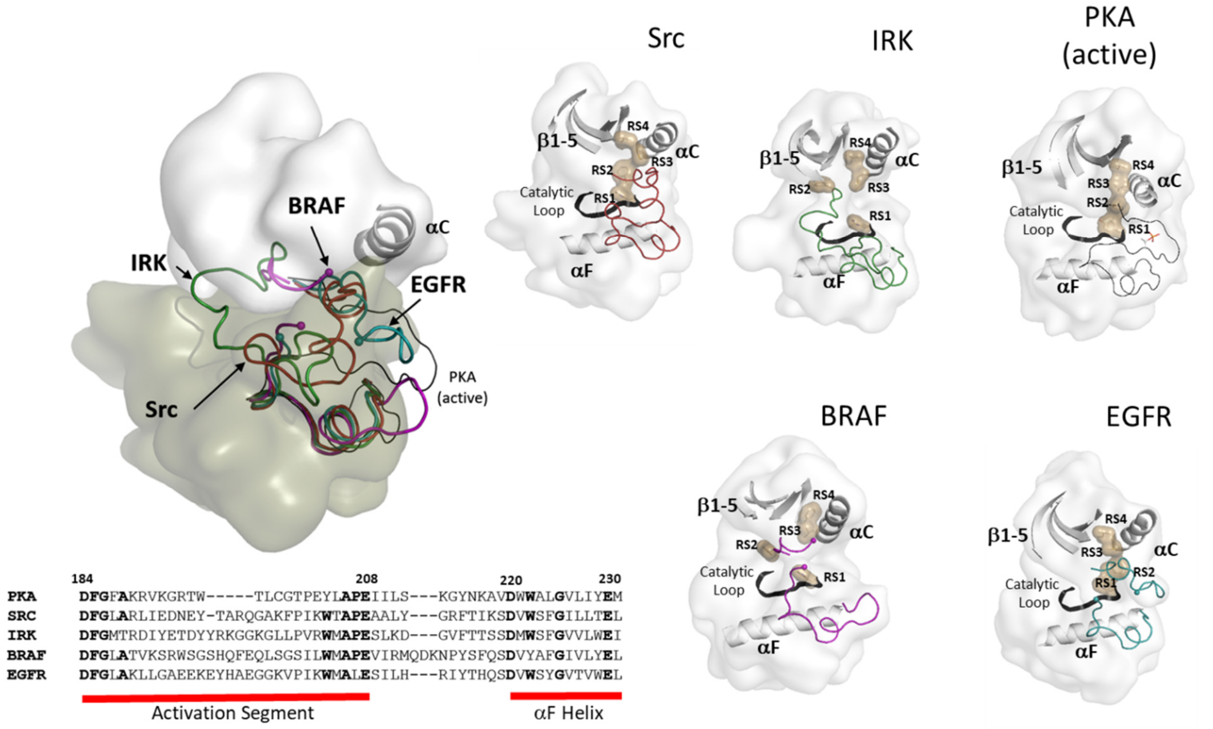


**Figure S6: Activation loops (ALs) of different kinases.** The AL orientations and broken R-spine conformations for inactive Src, IRK, BRAF and EGFR are compared to the AL and assembled R-spine of active PKA. The AL within the activation segment is a highly flexible part of the kinase domain and in most inactive kinases it is disordered. It connects several parts of the kinase core (e.g. αC helix, DFG motif) and is crucially involved in R-spine formation. For this, a specific, ordered orientation of the AL is important, which can be induced by phosphorylation (AL phosphorylation) or influenced by other domains which are not part of the kinase domain. The ordered orientation of the AL as shown above for PKA is mostly conserved for all protein kinases.

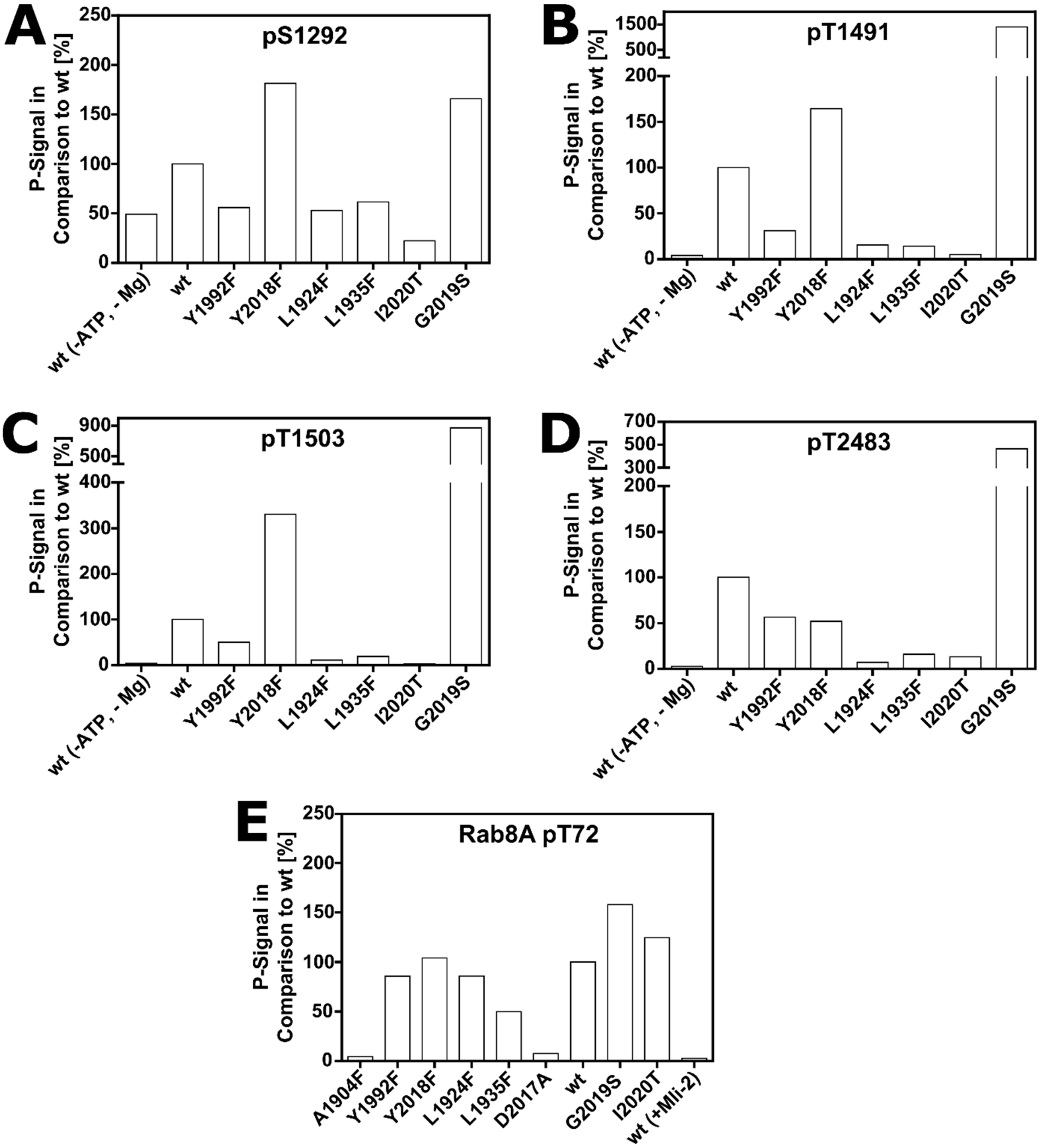


**Figure S7: LRRK2 auto- and substrate phosphorylation signals.** Western blots were prepared for the LRRK2 variants (see Fig.3). Therefore, the LRRK2 autophosphorylation signals (**A-D**) as well as the phosphorylation signals of Rab8A (**E**) by LRRK2 were quantified. Phosphorylation signals were normalized to the total LRRK2 or Rab8A protein signals and the phosphorylation signal of wt for either of the phosphorylation sites was set to 100 %.
